# Tetra­kis(μ-3-chloro­benzoato-κ^2^ *O*:*O*′)bis­[(*N*,*N*-di­ethyl­nicotinamide-κ*N* ^1^)copper(II)]

**DOI:** 10.1107/S1600536813017881

**Published:** 2013-07-03

**Authors:** Nihat Bozkurt, Tuncay Tunç, Nagihan Çaylak Delibaş, Hacali Necefoğlu, Tuncer Hökelek

**Affiliations:** a Department of Chemistry, Kafkas University, 36100 Kars, Turkey; b Aksaray University, Science Education Department, 68100, Aksaray, Turkey; c Department of Physics, Sakarya University, 54187 Esentepe, Sakarya, Turkey; d Department of Physics, Hacettepe University, 06800 Beytepe, Ankara, Turkey

## Abstract

In the title centrosymmetric binuclear Cu^II^ complex, [Cu_2_(C_7_H_4_ClO_2_)_4_(C_10_H_14_N_2_O)_2_], the two Cu^II^ cations [Cu⋯Cu = 2.6314 (4) Å] are bridged by four 3-chloro­benzoate (CB) anions. The four carboxyl­ate O atoms around each Cu^II^ cation form a distorted square-planar arrangement, the distorted square-pyramidal coordination geometry being completed by the pyridine N atom of the *N*,*N*-di­ethyl­nicotinamide (DENA) mol­ecule. The dihedral angle between the benzene ring and the carboxyl­ate group is 4.49 (11)° in one of the independent CB ligands and 12.00 (10)° in the other. The benzene rings of the independent CB ligands are oriented at a dihedral angle of 84.13 (6)°. In the crystal, weak C—H⋯O hydrogen bonds link the binuclear complex mol­ecules into supra­molecular chains running along [101].

## Related literature

For niacin, see: Krishnamachari (1974 ▶). For *N*,*N*-di­ethyl­nicotinamide, see: Bigoli *et al.* (1972 ▶). For related structures, see: Speier & Fulop (1989 ▶); Usubaliev *et al.* (1980 ▶); Hökelek *et al.* (1995 ▶); Hökelek *et al.* (2009*a* ▶,*b* ▶,*c* ▶, 2011 ▶); Necefoğlu *et al.* (2010**a* ▶,b* ▶); Aydın *et al.* (2012 ▶).
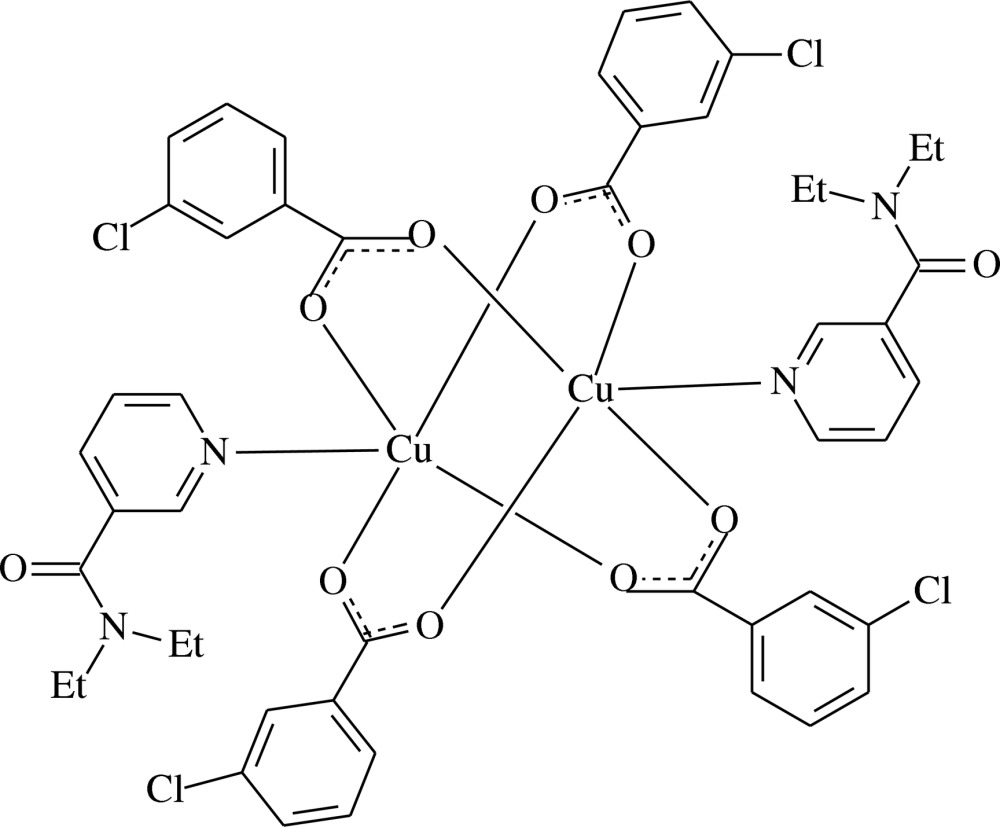


## Experimental

### 

#### Crystal data

[Cu_2_(C_7_H_4_ClO_2_)_4_(C_10_H_14_N_2_O)_2_]
*M* *_r_* = 1105.75
Monoclinic, 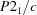

*a* = 12.6077 (4) Å
*b* = 16.7569 (5) Å
*c* = 12.1402 (4) Å
β = 99.647 (2)°
*V* = 2528.54 (14) Å^3^
*Z* = 2
Mo *K*α radiation
μ = 1.11 mm^−1^
*T* = 296 K
0.35 × 0.25 × 0.20 mm

#### Data collection

Bruker SMART BREEZE CCD diffractometer
Absorption correction: multi-scan (*SADABS*; Bruker, 2012 ▶) *T*_min_ = 0.711, *T*_max_ = 0.724
78293 measured reflections
5166 independent reflections
4702 reflections with *I* > 2σ(*I*)
*R*_int_ = 0.026

#### Refinement

*R*[*F* ^2^ > 2σ(*F* ^2^)] = 0.031
*wR*(*F* ^2^) = 0.083
*S* = 1.04
5166 reflections
309 parameters
85 restraints
H-atom parameters constrained
Δρ_max_ = 0.70 e Å^−3^
Δρ_min_ = −0.45 e Å^−3^

### 

Data collection: *APEX2* (Bruker, 2012 ▶); cell refinement: *SAINT* (Bruker, 2012 ▶); data reduction: *SAINT*; program(s) used to solve structure: *SHELXS97* (Sheldrick, 2008 ▶); program(s) used to refine structure: *SHELXL97* (Sheldrick, 2008 ▶); molecular graphics: *ORTEP-3 for Windows* (Farrugia, 2012 ▶); software used to prepare material for publication: *WinGX* (Farrugia, 2012 ▶) and *PLATON* (Spek, 2009 ▶).

## Supplementary Material

Crystal structure: contains datablock(s) I, global. DOI: 10.1107/S1600536813017881/xu5716sup1.cif

Structure factors: contains datablock(s) I. DOI: 10.1107/S1600536813017881/xu5716Isup2.hkl

Additional supplementary materials:  crystallographic information; 3D view; checkCIF report

## Figures and Tables

**Table 1:** Selected bond lengths (Å)

Cu1—O1	1.9791 (13)
Cu1—O2^i^	1.9688 (13)
Cu1—O3	1.9755 (13)
Cu1—O4^i^	1.9703 (13)
Cu1—N1	2.1454 (14)

Symmetry code: (i) 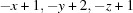.

**Table 2:** Hydrogen-bond geometry (Å, °)

*D*—H⋯*A*	*D*—H	H⋯*A*	*D*⋯*A*	*D*—H⋯*A*
C17—H17⋯O5^ii^	0.93	2.45	3.221 (3)	140

Symmetry code: (ii) 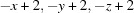.

**Fig. 1.**
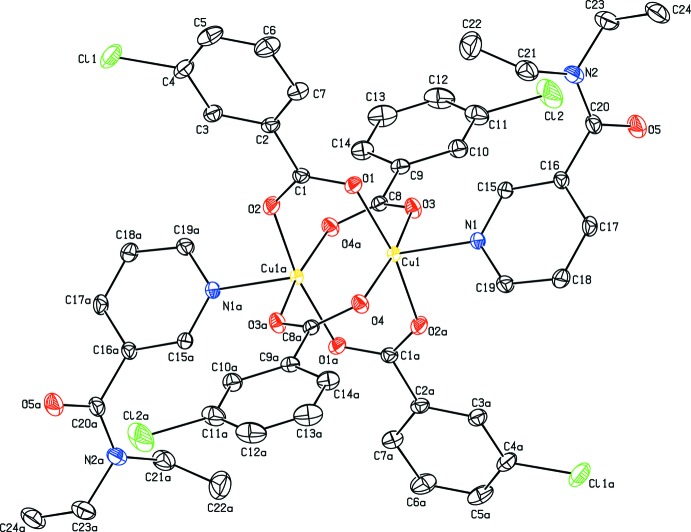
The molecular structure of the title molecule with the atom-numbering scheme. Displacement ellipsoids are drawn at the 50% probability level [symmetry code: (a) 1 - x, - y, 1 - z]. Hydrogen atoms have been omitted for clarity.

**Fig. 2.**
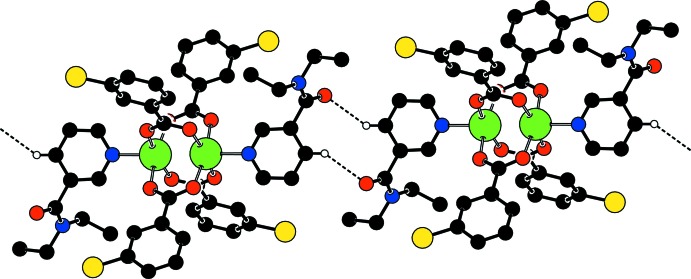
A view of the packing of the title compound. Hydrogen bonds are shown as dashed lines. Hydrogen atoms not involved in hydrogen bonding have been omitted for clarity.

## supplementary crystallographic information

### Comment

As a part of our ongoing investigation on transition metal complexes of nicotinamide (NA), one form of niacin (Krishnamachari, 1974), and/or the nicotinic acid derivative *N*,*N*-diethylnicotinamide (DENA), an important respiratory stimulant (Bigoli *et al.*, 1972), the title compound was synthesized and its crystal structure is reported herein.

The title compound is a binuclear compound, consisting of four chlorobenzoate (CB) ligands. The structures of similar complexes of the Cu^2+^, Zn^2+^ and Co^2+^ ions, [Cu(C_6_H_5_COO)_2_(C_5_H_5_N)]_2_ (Usubaliev *et al.*, 1980); [Cu(C_6_H_5_CO_2_)_2_(Py)]_2_ (Speier & Fulop, 1989); [Cu_2_(C_6_H_5_COO)_4_(C_10_H_14_N_2_O)_2_] (Hökelek *et al.*, 1995); [Cu_2_(C_8_H_7_O_2_)_4_(C_6_H_6_N_2_O)_2_] (Necefoğlu *et al.*, 2010*a*); [Cu_2_(C_7_H_4_IO_2_)_4_(H_2_O)_2_] (Aydın *et al.*, 2012); [Zn_2_(C_11_H_14_NO_2_)_4_(C_10_H_14_N_2_O)_2_] (Hökelek *et al.*, 2009*a*); [Zn_2_(C_8_H_8_NO_2_)_4_(C_10_H_14_N_2_O)_2_].2H_2_O (Hökelek *et al.*, 2009*b*); [Zn_2_(C_9_H_10_NO_2_)_4_(C_10_H_14_N_2_O)_2_] (Hökelek *et al.*, 2009*c*); [Zn_2_(C_8_H_7_O_2_)_4_(C_10_H_14_N_2_O)_2_] (Necefoğlu *et al.*, 2010*b*) and [Co_2_(C_11_H_14_NO_2_)_4_(C_10_H_14_N_2_O)_2_] (Hökelek *et al.*, 2011) have also been determined. In these structures, the benzoate ion acts as a bidentate ligand.

The title dimeric complex, [Cu_2_(CB)_4_(DENA)_2_], has a centre of symmetry and two Cu^II^ atoms are surrounded by four CB groups and two DENA molecules. The CB groups act as bridging ligands. The Cu1···Cu1a [symmetry code: (a) 1 - *x*, - *y*, 1 - *z*] distance is 2.6314 (4) Å. The average Cu-O distance is 1.9734 (13) Å (Table 1), and four O atoms of the bridging CB ligands around each Cu atom form a distorted square plane. The Cu atom lies 1.3997 (2) Å below the least-squares plane. The average O-Cu-O bond angle is 89.41 (6)°. A distorted square-pyramidal arrangement around each Cu atom is completed by the DENA N atom at 2.1454 (14) Å from the Cu atom (Table 1). The N1-Cu1···Cu1a angle is 173.85 (4)° and the dihedral angle between plane through Cu1, O1, O2, C1, Cu1a, O1a, O2a, C1a and the plane through Cu1, O3, O4, C8, Cu1a, O3a, O4a, C8a is 89.76(1.45)°. The dihedral angles between the planar carboxylate groups [(O1/O2/C1) and (O3/O4/C8)] and the adjacent benzene rings A (C2—C7) and B (C9—C14) are 4.49 (11) and 12.00 (10) °, respectively, while that between rings A, B and C (N1/C15–C19) are A/B = 84.13 (6), A/C = 57.94 (6) and B/C = 37.95 (7) °.

In the crystal structure, weak intermolecular C—H···O interactions (Table 2) link the molecules into infinite chains through the centrosymmetric dimers running along the [1 0 1] direction.

### Experimental

The title compound was prepared by the reaction of CuSO_4_.5H_2_O (1.25 g, 5 mmol) in H_2_O (100 ml) and diethylnicotinamide (1.78 g, 10 mmol) in H_2_O (20 ml) with sodium 3-chlorobenzoate (1.79 g, 10 mmol) in H_2_O (100 ml). The mixture was set aside to crystallize at ambient temperature for five days, giving green single crystals.

### Refinement

The C-bound H-atoms were positioned geometrically with C—H = 0.93, 0.97 and 0.96 Å, for aromatic, methylene and methyl H-atoms, respectively, and constrained to ride on their parent atoms, with *U*_iso_(H) = k × *U*_eq_(C), where k = 1.5 for methyl H-atoms and k = 1.2 for all other H-atoms.

### Crystal data

**Table:**

[Cu_2_(C_7_H_4_ClO_2_)_4_(C_10_H_14_N_2_O)_2_]	*F*(000) = 1132
*M**_r_* = 1105.75	*D*_x_ = 1.452 Mg m^−^^3^
Monoclinic, *P*2_1_/*c*	Mo *K*α radiation, λ = 0.71073 Å
Hall symbol: -P 2ybc	Cell parameters from 9380 reflections
*a* = 12.6077 (4) Å	θ = 2.5–29.9°
*b* = 16.7569 (5) Å	µ = 1.11 mm^−^^1^
*c* = 12.1402 (4) Å	*T* = 296 K
β = 99.647 (2)°	Block, green
*V* = 2528.54 (14) Å^3^	0.35 × 0.25 × 0.20 mm
*Z* = 2	

### Data collection

**Table:**

Bruker SMART BREEZE CCD diffractometer	5166 independent reflections
Radiation source: fine-focus sealed tube	4702 reflections with *I* > 2σ(*I*)
Graphite monochromator	*R*_int_ = 0.026
φ and ω scans	θ_max_ = 26.4°, θ_min_ = 1.6°
Absorption correction: multi-scan (*SADABS*; Bruker, 2012)	*h* = −15→15
*T*_min_ = 0.711, *T*_max_ = 0.724	*k* = −20→20
78293 measured reflections	*l* = −15→15

### Refinement

**Table:**

Refinement on *F*^2^	Primary atom site location: structure-invariant direct methods
Least-squares matrix: full	Secondary atom site location: difference Fourier map
*R*[*F*^2^ > 2σ(*F*^2^)] = 0.031	Hydrogen site location: inferred from neighbouring sites
*wR*(*F*^2^) = 0.083	H-atom parameters constrained
*S* = 1.04	*w* = 1/[σ^2^(*F*_o_^2^) + (0.0449*P*)^2^ + 1.301*P*] where *P* = (*F*_o_^2^ + 2*F*_c_^2^)/3
5166 reflections	(Δ/σ)_max_ = 0.002
309 parameters	Δρ_max_ = 0.70 e Å^−^^3^
85 restraints	Δρ_min_ = −0.45 e Å^−^^3^

### Special details

**Table:**

Geometry. All esds (except the esd in the dihedral angle between two l.s. planes) are estimated using the full covariance matrix. The cell esds are taken into account individually in the estimation of esds in distances, angles and torsion angles; correlations between esds in cell parameters are only used when they are defined by crystal symmetry. An approximate (isotropic) treatment of cell esds is used for estimating esds involving l.s. planes.
Refinement. Refinement of F^2^ against ALL reflections. The weighted R-factor wR and goodness of fit S are based on F^2^, conventional R-factors R are based on F, with F set to zero for negative F^2^. The threshold expression of F^2^ > 2sigma(F^2^) is used only for calculating R-factors(gt) etc. and is not relevant to the choice of reflections for refinement. R-factors based on F^2^ are statistically about twice as large as those based on F, and R- factors based on ALL data will be even larger.

### Fractional atomic coordinates and isotropic or equivalent isotropic displacement parameters (Å^2^)

**Table:**

	*x*	*y*	*z*	*U*_iso_*/*U*_eq_	
Cu1	0.580773 (15)	0.980853 (12)	0.578926 (15)	0.02838 (8)	
Cl1	0.53514 (7)	0.79998 (5)	−0.03442 (5)	0.0761 (2)	
Cl2	0.92843 (7)	1.31020 (5)	0.55213 (9)	0.1053 (3)	
O1	0.63814 (10)	0.91758 (8)	0.46442 (10)	0.0409 (3)	
O2	0.49984 (11)	0.95064 (9)	0.33241 (11)	0.0458 (3)	
O3	0.64805 (10)	1.07718 (8)	0.52693 (11)	0.0422 (3)	
O4	0.51122 (10)	1.10927 (8)	0.39242 (11)	0.0405 (3)	
O5	1.04739 (13)	1.05214 (11)	0.83585 (15)	0.0658 (5)	
N1	0.70894 (11)	0.96124 (10)	0.71646 (12)	0.0347 (3)	
N2	1.06037 (13)	0.95243 (12)	0.71450 (16)	0.0502 (4)	
C1	0.58602 (14)	0.91427 (10)	0.36697 (15)	0.0343 (4)	
C2	0.62814 (15)	0.86185 (11)	0.28384 (15)	0.0373 (4)	
C3	0.57253 (17)	0.85814 (12)	0.17511 (16)	0.0433 (4)	
H3	0.5123	0.8900	0.1529	0.052*	
C4	0.6080 (2)	0.80633 (14)	0.10029 (17)	0.0518 (5)	
C5	0.6965 (2)	0.75909 (16)	0.1303 (2)	0.0661 (7)	
H5	0.7186	0.7242	0.0790	0.079*	
C6	0.7521 (2)	0.76385 (16)	0.2371 (2)	0.0678 (7)	
H6	0.8127	0.7322	0.2581	0.081*	
C7	0.71904 (18)	0.81551 (13)	0.31447 (19)	0.0493 (5)	
H7	0.7579	0.8188	0.3865	0.059*	
C8	0.60290 (14)	1.12046 (10)	0.44801 (14)	0.0319 (3)	
C9	0.66516 (15)	1.19115 (11)	0.41815 (16)	0.0368 (4)	
C10	0.75758 (17)	1.21469 (12)	0.48927 (19)	0.0463 (5)	
H10	0.7814	1.1867	0.5549	0.056*	
C11	0.81362 (19)	1.28015 (14)	0.4613 (2)	0.0620 (6)	
C12	0.7809 (2)	1.32217 (15)	0.3639 (3)	0.0749 (8)	
H12	0.8202	1.3659	0.3461	0.090*	
C13	0.6896 (2)	1.29872 (15)	0.2938 (3)	0.0700 (7)	
H13	0.6668	1.3267	0.2280	0.084*	
C14	0.63081 (19)	1.23348 (13)	0.32036 (19)	0.0506 (5)	
H14	0.5685	1.2182	0.2727	0.061*	
C15	0.81050 (14)	0.97600 (11)	0.70628 (15)	0.0361 (4)	
H15	0.8250	0.9911	0.6366	0.043*	
C16	0.89570 (14)	0.97002 (11)	0.79394 (15)	0.0353 (4)	
C17	0.87265 (15)	0.94853 (12)	0.89783 (15)	0.0412 (4)	
H17	0.9273	0.9452	0.9595	0.049*	
C18	0.76805 (17)	0.93223 (14)	0.90842 (16)	0.0468 (5)	
H18	0.7512	0.9167	0.9770	0.056*	
C19	0.68845 (15)	0.93925 (13)	0.81604 (16)	0.0425 (4)	
H19	0.6178	0.9282	0.8237	0.051*	
C20	1.00821 (15)	0.99464 (13)	0.78250 (17)	0.0429 (4)	
C21	1.02241 (19)	0.87796 (16)	0.6595 (2)	0.0649 (7)	
H211	0.9638	0.8574	0.6937	0.078*	
H212	1.0803	0.8391	0.6717	0.078*	
C22	0.9840 (3)	0.8867 (3)	0.5347 (3)	0.1069 (13)	
H221	0.9232	0.9220	0.5219	0.160*	
H222	0.9635	0.8354	0.5030	0.160*	
H223	1.0410	0.9083	0.5003	0.160*	
C23	1.17124 (19)	0.97631 (17)	0.7067 (2)	0.0660 (7)	
H231	1.1878	0.9589	0.6353	0.079*	
H232	1.1761	1.0341	0.7092	0.079*	
C24	1.25301 (19)	0.9423 (2)	0.7983 (3)	0.0759 (8)	
H241	1.2468	0.8852	0.7983	0.114*	
H242	1.3238	0.9570	0.7864	0.114*	
H243	1.2409	0.9630	0.8689	0.114*	

### Atomic displacement parameters (Å^2^)

**Table:**

	*U*^11^	*U*^22^	*U*^33^	*U*^12^	*U*^13^	*U*^23^
Cu1	0.02465 (11)	0.03345 (13)	0.02565 (12)	−0.00047 (7)	0.00020 (8)	−0.00112 (8)
Cl1	0.1050 (5)	0.0886 (5)	0.0378 (3)	−0.0216 (4)	0.0206 (3)	−0.0159 (3)
Cl2	0.0775 (5)	0.0876 (5)	0.1497 (8)	−0.0475 (4)	0.0157 (5)	−0.0400 (6)
O1	0.0411 (7)	0.0475 (8)	0.0346 (7)	0.0051 (6)	0.0077 (5)	−0.0066 (6)
O2	0.0428 (7)	0.0565 (8)	0.0370 (7)	0.0102 (6)	0.0033 (6)	−0.0131 (6)
O3	0.0388 (7)	0.0412 (7)	0.0432 (7)	−0.0097 (6)	−0.0030 (5)	0.0079 (6)
O4	0.0352 (7)	0.0427 (7)	0.0420 (7)	−0.0059 (5)	0.0018 (5)	0.0078 (6)
O5	0.0505 (9)	0.0705 (11)	0.0739 (11)	−0.0157 (8)	0.0032 (8)	−0.0230 (9)
N1	0.0292 (7)	0.0439 (8)	0.0293 (7)	0.0024 (6)	0.0000 (6)	0.0020 (6)
N2	0.0353 (8)	0.0583 (11)	0.0583 (11)	−0.0018 (8)	0.0114 (7)	−0.0050 (9)
C1	0.0339 (9)	0.0340 (9)	0.0370 (9)	−0.0054 (7)	0.0115 (7)	−0.0037 (7)
C2	0.0426 (10)	0.0342 (9)	0.0386 (9)	−0.0065 (7)	0.0175 (8)	−0.0034 (7)
C3	0.0485 (11)	0.0447 (11)	0.0396 (10)	−0.0073 (9)	0.0152 (8)	−0.0041 (8)
C4	0.0713 (14)	0.0516 (12)	0.0372 (10)	−0.0171 (11)	0.0227 (10)	−0.0097 (9)
C5	0.0912 (19)	0.0568 (14)	0.0586 (14)	0.0074 (13)	0.0371 (13)	−0.0140 (12)
C6	0.0772 (17)	0.0645 (16)	0.0668 (15)	0.0230 (13)	0.0275 (13)	−0.0051 (13)
C7	0.0532 (12)	0.0502 (12)	0.0475 (11)	0.0052 (9)	0.0175 (9)	−0.0023 (9)
C8	0.0336 (8)	0.0320 (8)	0.0313 (8)	−0.0012 (7)	0.0089 (7)	−0.0025 (7)
C9	0.0388 (9)	0.0325 (9)	0.0422 (10)	−0.0010 (7)	0.0160 (7)	−0.0024 (7)
C10	0.0464 (11)	0.0413 (10)	0.0532 (12)	−0.0095 (9)	0.0141 (9)	−0.0081 (9)
C11	0.0547 (13)	0.0458 (12)	0.0905 (18)	−0.0178 (10)	0.0269 (12)	−0.0182 (12)
C12	0.0827 (19)	0.0418 (12)	0.112 (2)	−0.0138 (12)	0.0507 (17)	0.0072 (14)
C13	0.0880 (19)	0.0502 (14)	0.0798 (18)	0.0066 (13)	0.0367 (15)	0.0238 (13)
C14	0.0570 (13)	0.0455 (11)	0.0523 (12)	0.0032 (9)	0.0178 (10)	0.0091 (9)
C15	0.0328 (9)	0.0463 (10)	0.0284 (8)	0.0019 (7)	0.0030 (7)	0.0019 (7)
C16	0.0298 (8)	0.0374 (9)	0.0368 (9)	0.0048 (7)	−0.0002 (7)	−0.0041 (7)
C17	0.0390 (10)	0.0477 (11)	0.0323 (9)	0.0063 (8)	−0.0071 (7)	0.0005 (8)
C18	0.0478 (11)	0.0610 (13)	0.0305 (9)	0.0047 (9)	0.0032 (8)	0.0074 (9)
C19	0.0341 (9)	0.0564 (12)	0.0366 (9)	−0.0003 (8)	0.0053 (7)	0.0065 (9)
C20	0.0325 (9)	0.0496 (11)	0.0430 (10)	0.0018 (8)	−0.0034 (8)	−0.0001 (9)
C21	0.0471 (12)	0.0638 (15)	0.0857 (17)	0.0030 (11)	0.0162 (12)	−0.0228 (13)
C22	0.087 (2)	0.141 (3)	0.086 (2)	0.022 (2)	−0.0043 (18)	−0.052 (2)
C23	0.0429 (12)	0.0784 (18)	0.0805 (18)	−0.0069 (11)	0.0214 (12)	0.0006 (14)
C24	0.0368 (12)	0.092 (2)	0.100 (2)	0.0002 (12)	0.0133 (12)	−0.0083 (17)

### Geometric parameters (Å, º)

**Table:**

Cu1—Cu1^i^	2.6314 (4)	C9—C10	1.386 (3)
Cu1—O1	1.9791 (13)	C9—C14	1.388 (3)
Cu1—O2^i^	1.9688 (13)	C10—C11	1.378 (3)
Cu1—O3	1.9755 (13)	C10—H10	0.9300
Cu1—O4^i^	1.9703 (13)	C11—C12	1.378 (4)
Cu1—N1	2.1454 (14)	C12—H12	0.9300
Cl1—C4	1.740 (2)	C13—C12	1.369 (4)
Cl2—C11	1.741 (3)	C13—H13	0.9300
O1—C1	1.255 (2)	C14—C13	1.388 (3)
O2—C1	1.255 (2)	C14—H14	0.9300
O2—Cu1^i^	1.9688 (13)	C15—H15	0.9300
O3—C8	1.259 (2)	C16—C15	1.383 (2)
O4—Cu1^i^	1.9703 (13)	C16—C20	1.506 (3)
O4—C8	1.251 (2)	C17—C16	1.388 (3)
O5—C20	1.218 (3)	C17—C18	1.374 (3)
N1—C15	1.330 (2)	C17—H17	0.9300
N1—C19	1.330 (2)	C18—H18	0.9300
N2—C20	1.340 (3)	C19—C18	1.379 (3)
N2—C21	1.458 (3)	C19—H19	0.9300
N2—C23	1.472 (3)	C21—C22	1.519 (4)
C1—C2	1.500 (2)	C21—H211	0.9700
C2—C3	1.388 (3)	C21—H212	0.9700
C2—C7	1.383 (3)	C22—H221	0.9600
C3—C4	1.384 (3)	C22—H222	0.9600
C3—H3	0.9300	C22—H223	0.9600
C4—C5	1.367 (4)	C23—C24	1.496 (4)
C5—C6	1.369 (4)	C23—H232	0.9700
C5—H5	0.9300	C23—H231	0.9700
C6—H6	0.9300	C24—H241	0.9600
C7—C6	1.392 (3)	C24—H242	0.9600
C7—H7	0.9300	C24—H243	0.9600
C8—C9	1.499 (2)		
			
O1—Cu1—Cu1^i^	87.05 (4)	C10—C11—Cl2	118.7 (2)
O1—Cu1—N1	98.53 (6)	C10—C11—C12	121.7 (2)
O2^i^—Cu1—Cu1^i^	81.29 (4)	C12—C11—Cl2	119.57 (19)
O2^i^—Cu1—O1	168.33 (6)	C11—C12—H12	120.5
O2^i^—Cu1—O3	89.25 (6)	C13—C12—C11	119.1 (2)
O2^i^—Cu1—O4^i^	88.70 (6)	C13—C12—H12	120.5
O2^i^—Cu1—N1	93.11 (6)	C12—C13—C14	120.5 (3)
O3—Cu1—Cu1^i^	83.65 (4)	C12—C13—H13	119.8
O3—Cu1—O1	89.21 (6)	C14—C13—H13	119.8
O3—Cu1—N1	93.79 (6)	C9—C14—C13	119.9 (2)
O4^i^—Cu1—Cu1^i^	84.77 (4)	C9—C14—H14	120.0
O4^i^—Cu1—O1	90.49 (6)	C13—C14—H14	120.0
O4^i^—Cu1—O3	168.41 (5)	N1—C15—C16	123.41 (17)
O4^i^—Cu1—N1	97.71 (6)	N1—C15—H15	118.3
N1—Cu1—Cu1^i^	173.85 (4)	C16—C15—H15	118.3
C1—O1—Cu1	119.39 (12)	C15—C16—C17	117.64 (17)
C1—O2—Cu1^i^	126.70 (12)	C15—C16—C20	122.09 (17)
C8—O3—Cu1	123.38 (11)	C17—C16—C20	119.91 (16)
C8—O4—Cu1^i^	122.52 (12)	C16—C17—H17	120.4
C15—N1—Cu1	120.69 (12)	C18—C17—C16	119.14 (16)
C19—N1—Cu1	120.94 (12)	C18—C17—H17	120.4
C19—N1—C15	118.21 (15)	C17—C18—C19	119.09 (18)
C20—N2—C21	125.21 (18)	C17—C18—H18	120.5
C20—N2—C23	117.7 (2)	C19—C18—H18	120.5
C21—N2—C23	116.48 (19)	N1—C19—C18	122.49 (18)
O1—C1—O2	125.52 (16)	N1—C19—H19	118.8
O1—C1—C2	118.14 (16)	C18—C19—H19	118.8
O2—C1—C2	116.33 (16)	O5—C20—N2	123.2 (2)
C3—C2—C1	119.11 (17)	O5—C20—C16	118.15 (19)
C7—C2—C1	121.25 (17)	N2—C20—C16	118.64 (18)
C7—C2—C3	119.61 (18)	N2—C21—C22	113.6 (3)
C2—C3—H3	120.5	N2—C21—H211	108.9
C4—C3—C2	119.0 (2)	N2—C21—H212	108.9
C4—C3—H3	120.5	C22—C21—H211	108.9
C3—C4—Cl1	118.5 (2)	C22—C21—H212	108.9
C5—C4—Cl1	119.61 (17)	H211—C21—H212	107.7
C5—C4—C3	121.8 (2)	C21—C22—H221	109.5
C4—C5—C6	119.0 (2)	C21—C22—H222	109.5
C4—C5—H5	120.5	C21—C22—H223	109.5
C6—C5—H5	120.5	H221—C22—H222	109.5
C5—C6—C7	120.8 (2)	H221—C22—H223	109.5
C5—C6—H6	119.6	H222—C22—H223	109.5
C7—C6—H6	119.6	N2—C23—C24	113.1 (2)
C2—C7—C6	119.8 (2)	N2—C23—H232	109.0
C2—C7—H7	120.1	N2—C23—H231	109.0
C6—C7—H7	120.1	C24—C23—H232	109.0
O3—C8—C9	116.81 (15)	C24—C23—H231	109.0
O4—C8—O3	125.68 (16)	H232—C23—H231	107.8
O4—C8—C9	117.52 (16)	C23—C24—H243	109.5
C10—C9—C8	119.60 (18)	C23—C24—H242	109.5
C10—C9—C14	119.75 (19)	C23—C24—H241	109.5
C14—C9—C8	120.64 (18)	H243—C24—H242	109.5
C9—C10—H10	120.5	H243—C24—H241	109.5
C11—C10—C9	119.0 (2)	H242—C24—H241	109.5
C11—C10—H10	120.5		
			
Cu1^i^—Cu1—O1—C1	−1.49 (13)	O1—C1—C2—C3	−179.12 (17)
O2^i^—Cu1—O1—C1	−2.7 (4)	O1—C1—C2—C7	2.9 (3)
O3—Cu1—O1—C1	−85.17 (14)	O2—C1—C2—C3	1.7 (3)
O4^i^—Cu1—O1—C1	83.25 (14)	O2—C1—C2—C7	−176.27 (18)
N1—Cu1—O1—C1	−178.88 (13)	C1—C2—C3—C4	−176.33 (17)
Cu1^i^—Cu1—O3—C8	−0.31 (14)	C7—C2—C3—C4	1.7 (3)
O1—Cu1—O3—C8	86.81 (15)	C3—C2—C7—C6	−1.9 (3)
O2^i^—Cu1—O3—C8	−81.63 (15)	C1—C2—C7—C6	176.0 (2)
O4^i^—Cu1—O3—C8	−1.8 (4)	C2—C3—C4—Cl1	178.00 (15)
N1—Cu1—O3—C8	−174.70 (14)	C2—C3—C4—C5	−0.4 (3)
O1—Cu1—N1—C15	52.96 (15)	Cl1—C4—C5—C6	−179.0 (2)
O1—Cu1—N1—C19	−131.61 (16)	C3—C4—C5—C6	−0.7 (4)
O2^i^—Cu1—N1—C15	−126.27 (15)	C4—C5—C6—C7	0.4 (4)
O2^i^—Cu1—N1—C19	49.16 (16)	C2—C7—C6—C5	0.9 (4)
O3—Cu1—N1—C15	−36.82 (15)	O3—C8—C9—C10	−12.2 (2)
O3—Cu1—N1—C19	138.61 (16)	O3—C8—C9—C14	168.16 (18)
O4^i^—Cu1—N1—C15	144.62 (14)	O4—C8—C9—C10	168.11 (17)
O4^i^—Cu1—N1—C19	−39.95 (16)	O4—C8—C9—C14	−11.5 (3)
Cu1—O1—C1—O2	2.8 (3)	C8—C9—C10—C11	−179.51 (18)
Cu1—O1—C1—C2	−176.35 (11)	C14—C9—C10—C11	0.1 (3)
Cu1^i^—O2—C1—O1	−2.6 (3)	C8—C9—C14—C13	−179.86 (19)
Cu1^i^—O2—C1—C2	176.52 (12)	C10—C9—C14—C13	0.5 (3)
Cu1—O3—C8—O4	0.1 (3)	C9—C10—C11—Cl2	179.02 (16)
Cu1—O3—C8—C9	−179.54 (11)	C9—C10—C11—C12	−0.7 (3)
Cu1^i^—O4—C8—O3	0.3 (3)	C10—C11—C12—C13	0.7 (4)
Cu1^i^—O4—C8—C9	179.96 (11)	Cl2—C11—C12—C13	−179.1 (2)
Cu1—N1—C15—C16	175.23 (14)	C14—C13—C12—C11	0.0 (4)
C19—N1—C15—C16	−0.3 (3)	C9—C14—C13—C12	−0.6 (4)
Cu1—N1—C19—C18	−174.76 (16)	C17—C16—C15—N1	−0.9 (3)
C15—N1—C19—C18	0.8 (3)	C20—C16—C15—N1	−174.04 (18)
C21—N2—C20—O5	172.2 (2)	C15—C16—C20—O5	113.2 (2)
C21—N2—C20—C16	−7.7 (3)	C15—C16—C20—N2	−66.8 (3)
C23—N2—C20—O5	1.4 (3)	C17—C16—C20—O5	−59.7 (3)
C23—N2—C20—C16	−178.50 (19)	C17—C16—C20—N2	120.2 (2)
C20—N2—C21—C22	107.6 (3)	C18—C17—C16—C15	1.7 (3)
C23—N2—C21—C22	−81.5 (3)	C18—C17—C16—C20	174.99 (19)
C20—N2—C23—C24	83.6 (3)	C16—C17—C18—C19	−1.3 (3)
C21—N2—C23—C24	−88.0 (3)	N1—C19—C18—C17	0.0 (3)

Symmetry code: (i) −*x*+1, −*y*+2, −*z*+1.

### Hydrogen-bond geometry (Å, º)

**Table:**

*D*—H···*A*	*D*—H	H···*A*	*D*···*A*	*D*—H···*A*
C17—H17···O5^ii^	0.93	2.45	3.221 (3)	140

Symmetry code: (ii) −*x*+2, −*y*+2, −*z*+2.
